# Easter Day, 1916

**Published:** 1916-04-22

**Authors:** 


### THE MATRONS' AND SISTERS' DEPARTMENT.

### EASTER DAY, 1916.

*"The Care of the Sick is the object of Hospitals."—Florence Nightingale.*

Easter Day 1869 was the first occasion in his life on which the Editor attended Easter service in the chapel of a hospital as its superintendent. The occasion was memorable from the circumstance that it was the first Easter Sunday in Birmingham when a hospital chapel had amongst its congregation probationer nurses. Their presence commemorated a great change in the nursing world of those days, for it was in 1869 that probationer nurses were received for training, which included lectures by the resident and honorary medical officers, practical teaching in the wards with an examination which, if passed, would entitle the probationer to receive a certificate of one year's training and efficiency. In those days it was not easy, if it was even possible, to obtain suitable young women to be trained as hospital nurses. Mrs. Wardroper, in conjunction with the Editor, devised measures to secure suitable applicants, but the first year's results were that at St. Thomas's Hospital four, and at Birmingham only two out of 100 applicants in each case survived the test and became trained nurses.

Easter has recalled these early difficulties of the pioneers of trained nursing in England and created a feeling of thankfulness for the successful incorporation of the College of Nursing, which has proved a phenomenal success, as the names of those present at the meeting at St. Thomas's Hospital, given on page 63 of The Hospital of 15th instant, strikingly demonstrate. How many matrons, sisters, and trained nurses throughout the country next Sunday will thank God while they realise the wonderful developments in the nursing world since 1869! These include an agreement by all parties and all sections to three fundamental principles, State registration, a uniform curriculum, and one standardised examination for nurses. Further, is there one single disinterested well-wisher of British nurses and British nursing who is not in favour of these fundamentals, and the establishment of a College of Nursing? Easter Day 1916 should commemorate the unity, security, and direct representation of trained nurses everywhere, which the Council of the College has the noble ambition to promptly organise and make effective, as a living force.

Every thoughtful member of the nursing profession, and every matron and sister especially, will share our rejoicing that the College of Nursing should have been founded in 1916, the critical year of the Great War. This war has knit together all classes of the community, and in no centre of human life have its tragedies, with its blessings and teachings, been more keenly realised and driven home, than in the hospitals. Where else will Easter Day have a more heart-stirring influence, for there all who have had to do with the wounded will recall manifold incidents and circumstances, where Christ's teaching and example have raised the patients and their attendants far above the pain and the miseries of daily life, all earthly trials, sufferings, and death? The war has brought many partings and separations. It has removed a multitude of dear ones from the home and the family, and severed all earthly ties. Yet it has awakened throughout the nation in every centre of population, and in a multitude of homes, the grateful recognition of God's mercy and teaching, and the realisation of a higher and better life. The personal service of the workers in a hospital amongst the wounded from the battlefields, their intercourse with the warriors' relatives and friends, the constant and ever-changing incidents in the day's work, have knit together the staff, and especially the nursing staff, who have been inspired by the wish to give more and more personal service, with results, which, as we said recently, have become historical, and must remain memorable for all time.

April 23, 1916, Easter Day, St. George's Day, will be surely a golden day, which British matrons, sisters, and nurses will remember and thank God for. Their profession has come to occupy a position, which has placed it on the high-road to State recognition and a Royal College of Nursing. The war has opened to each one of them great opportunities for personal service for their country, the nation's warriors, and the uplifting of humanity. Who amongst them, that has spent many days since the war in attendance on the wounded, wherever they may have been placed, whether at the Front, at the base hospital, in the Motherland, or at outlying portions of the Empire, can ever forget what they have seen and what they have been privileged to accomplish? Just as the war has aroused the highest manhood of the nation and empire, so it has called forth everything that is best, in the best women of our race. Who amongst us has not learnt again with gratitude, the abiding truths of Christianity and their uplifting influence for the individual worker, however trying the circumstances and however difficult the task? English families, when widely separated, are accustomed to unite at the Altar at a given hour on Easter morning. We believe that all workers amongst the sick will resolve to assemble there on Easter Day 1916. The opportunities enjoyed, the good work accomplished, the blessings experienced, and the splendid outlook for the whole nursing profession which is now attained, call for praise, for praise is the one unselfish act which the created can render to the Creator.

**MATRONS' AND SISTERS' DEPARTMENT—(*continued*).**

The maintenance of order in out-patients' departments of hospitals is not always an easy matter. The desire to be attended to quickly, and the fear of each individual that he may lose his turn, make it difficult to prevent the patients from crowding round the door of the consulting-room. A method of ensuring the applicants being dealt with in turn is in use at the British Consulate in Paris, where large numbers of people are often assembled to wait for their passports to be *viséd*, which is so simple and effective that it might be adopted with advantage in hospital work. A block of about 500 numbered slips of paper is hung in a prominent position just below a large notice bearing the legend "Please tear off a number." The first-comer naturally gets No. 1, and the second arrival No. 2, and so on, with the result that the patients sit down where they are told, knowing that only the possessor of the following number will be admitted next to the consulting-room. The reverse side of the slip can be used by the nurse for recording the patient's temperature.

"I'm sorry to hear your son is in hospital, Mrs. Jones; I hope it is nothing serious." "Well, ma'am, I'm rather afeard he must be pretty bad." "Did not he say what was the matter?" "That's just what's worrying me; I got word as he was wounded in the dardanelles, and I don't know what part of the body they lies in. It sounds serious-like, and I'm thinking as it must be one of them internal troubles, to which the doctors have give a name as nobody can contradict."

